# Editorial: Biofunctional Molecule Exploratory Research on Application in Food and Health

**DOI:** 10.3390/molecules28135089

**Published:** 2023-06-29

**Authors:** Wei Hsum Yap, Bey Hing Goh

**Affiliations:** 1 School of Biosciences, Faculty of Medical and Health Sciences, Taylor’s University, Subang Jaya 47500, Malaysia; 2 Centre for Drug Discovery and Molecular Pharmacology, Faculty of Medical and Health Sciences, Taylor’s University, Subang Jaya 47500, Malaysia; 3 Biofunctional Molecule Exploratory (BMEX) Research Group, School of Pharmacy, Monash University Malaysia, Bandar Sunway 47500, Malaysia; 4 College of Pharmaceutical Sciences, Zhejiang University, Hangzhou 310058, China

## 1. Introduction

Biofunctional molecules with pharmacological activities are reported in various fields of application, including in the pharmaceutical, cosmetics, nutraceuticals, agriculture, and food industries. Naturally derived bioactive compounds may be developed as therapeutic agents, functional foods, biosurfactants, feed additives, or cosmetical formulations. Bioactive molecules could be utilized as extracts, fractions, or isolated pure compounds, where they exhibit various biological effects, including the modulation of specific protein targets such as enzymes, ion channels, and receptors, as well as regulating cellular metabolism with a beneficial response. The use of biofunctional molecules and extracts has been associated with the maintenance and promotion of human health through the prevention and treatment of diseases. The Special Issue “Biofunctional Molecule Exploratory Research on Application in Food and Health” aimed to emphasize the value of natural products and their associated bioactive molecules, which can be used for the development of nutraceutical supplements and as potential cosmeceutical applications.

## 2. Natural-Product-Based Extracts and Their Functional Properties

Herein, Mahleyuddin et al. [1] have reported the cardioprotective benefits of *Coriandrum sativum* L., a plant belonging to the Apiaceae (Umbelliferae) family, which is recognized for its use in culinary and traditional purposes. Its cardioprotective benefits have been described in numerous preclinical animal model studies, which showed potential in inducing antioxidative and antiatherogenic properties, hypolipidemic activity, antihypertensive potential, and antiarrhythmic activity. Notably, the *C. sativum* extract contained active phytochemicals, including flavonoids, phenolic acids, and phytosterols, which could contribute to its cardioprotective properties.

Meanwhile, Tchetan et al. [2] have identified several bioactive phytochemical compounds from *Terminalia leiocarpa* (DC.) Baill that pose significant anthelmintic activity. *Terminalia leiocarpa*, a type of tree found in India and Africa, has been widely reported for its use as traditional medicine, as wood, and in energy production. The research group confirmed that both ellagic and gallic acids, the major compounds isolated from *Terminalia leiocarpa*, were the most effective at inhibiting *Caenorhabditis elegans* viability. Nevertheless, it is argued that an additive/synergistic effect of the different compounds present in the extracts contributes to the anthelmintic activity.

Ethnobotanical investigations have shown that *Tamarindus indica* and *Mitragyna inermis* are beneficial for the treatment of diabetes mellitus. Herein, Ouédraogo et al. [3] indicated that active fractions from these two extracts showed significant antiglycation activity. The team also showed that all fractions presented no adverse effects on NIH-3T3 up to 500 µg/mL. The isolated pure compounds from these fractions, including sweroside and triterpenoids, however, were found to be inactive when tested for the inhibition of fructose-mediated BSA glycation.

## 3. Active Pharmacological Constituents and Their Derivates for Potential Pharmaceutical Applications

Ayoub and team have synthesized two benzoxazole derivatives, namely 2-(4-Chlorophenyl)-5-benzoxazoleacetic acid (CBA) and its prodrug, methyl-2-(4-chloro-phenyl)-5-benzoxazoleacetate (MCBA), and reported its efficacy on imiquimod (IMQ)-induced psoriatic mouse model [4]. The study proved that CBA and MCBA can inhibit psoriasis-associated inflammatory symptoms, including decreases in erythema intensity, thickness, and desquamation, as well as reductions in hyperkeratosis and hyperplasia. Notably, these effects were comparable with the reference drug clobetasol propionate. Interestingly, the protective effects against psoriasis were found to be stronger in MCBA-treated mice than in CBA-treated mice. Hence, the group proposed that the development of a prodrug could potentially enhance the antipsoriatic effects.

Meanwhile, the 2,3-Dihydroindoles represent a group of promising agents that exhibit neuroprotective and antioxidant properties. Herein, Volkova et al. have reported the synthesis of new 2,3-dihydroindole derivatives as potential melatonin receptor ligands [5]. The research team used several boron hydrides to reduce the functional groups within 2-oxindole and 2-chloroindole molecules, and they proposed that a NaBH_4_/NiCl_2_ reagent led to cyano-group reduction while BH_3_-based reagents produced simultaneous reductions in both cyano- and amido-groups of the oxindole ring. The results demonstrated that the presence of the sp^3^ carbon at position 3 of the indole ring resulted in a reduction in melatonin receptor binding affinity, while lipophilic substituents in position 2 of the melatonin ring can increase the binding affinity to melatonin receptors. Volkova’s group proposed that the synthesis method can be utilized for producing new analogs of and other compounds with neuroprotective properties.

On the other hand, Liu et al. have shown that Kawain, a main kavalactone in the kava plant, exhibits chemopreventive effects against UPII-mutant Ha-Ras transgenic mice [6]. The research findings showed that Kawain enhanced survival and delayed the progression from hyperplasia to papillary carcinoma in non-muscle invasive urothelial tumor-bearing mice, as well as reducing urothelial cell carcinoma-induced hydronephrosis and hematuria. It is proposed that Kawain regulates the mTOR signaling pathway and inhibits bladder cancer cells’ growth.

Naturally occurring pentacyclic triterpenoid compounds are known to exhibit various beneficial properties, including antioxidant, anti-inflammatory, antimicrobial, and antitumor activities. Herein, Zong and team have shown that ursolic acid suppressed the adhesion and migration of doxorubicin-resistant breast cancer MCF7/ADR cells [7]. It was demonstrated that ursolic acid can bind to the active site of ornithine decarboxylase and downregulate its expression, as well as modulate other key tumorigenesis signaling factors, including phosphorylated Erk (P-Erk), VEGF, and matrix metalloproteinase-9 (MMP-9) activity. Notably, ursolic acid increased the intracellular accumulation of Dox in MCF-7/ADR cells, suggesting that it could regulate tumor progression during the treatment of breast cancer with doxorubicin.

## 4. Functional Foods as Natural Health-Promoting Nutritional Supplements

Functional foods containing antioxidant bioactive compounds may serve as potential nutritional supplements for the prevention and treatment of chronic diseases. Herein, Tsong and colleagues reviewed the phytochemical compositions and biological activities of *Nephelium lappaceum* and *Nephelium ramboutan-ake*, types of tropical fruit from the *Sapindaceae* family [8]. Interestingly, phenolics from these two plants served as potential antioxidants that contributed to antiaging, antineoplastic, antimicrobial, and hypoglycemic activities in in vitro and in vivo disease models. It is worth noting that the seeds and fruit peels for both plants contained more nutraceutical potential compared to the edible parts. Tsong et al. highlighted that more investigations are warranted for *Nephelium ramboutan-ake*, as knowledge on their active constituents and biological activities is still lacking. 

On the other hand, Moshawih et al. have evaluated the pharmacological activities of *Triticum aestivum*, more commonly known as wheat, the widely consumed cereal grain [9]. The review showed that the diverse components of wheat, including dietary fiber, resistant starch, phenolic acids, alkylresorcinols, lignans, and tocols, exhibit protective effects in the gastrointestinal tract, as well as anticancer, antimicrobial, cholesterol-lowering, phytoestrogen, and immune-modulatory activities. 

## 5. Applications of Biomolecules in the Agricultural and Food Processing Industries

In addition to exhibiting potential pharmacological activities, biomolecules are also applied in other sectors, including the agricultural and food processing industries. Cho et al. investigated the potential of sophorolipids, a natural glycolipid biosurfactant synthesized by yeast, in various applications in the food processing, agricultural, and healthcare industries [10]. Sophorolipids were gaining increasing interest due to their structural diversity and low-cost fermentative production. The review by Cho et al. showed that sophorolipids and their derivatives are utilized in food preservation, the bioconversion of food waste, the enhancement of plant growth, wound healing, and as part of antimicrobial formulating agents.

Meanwhile, the metabolic engineering of glutamate dehydrogenase (GDH) genes from *Pyropia haitanensis*, a type of red algae species, may provide insights on GDH enzyme activity and its glutamic acid metabolism, which contributes to its nutritional value and flavor. Herein, Li et al. have shown that *PhGDH1* and *PhGDH2*, two *GDH* genes from *P. haitanensis*, have differential *K_m_* values towards various substrates, such as NADH, (NH_4_)_2_SO_4_, and α-oxoglutarate [11]. It was shown that both enzymes have higher affinity for NH_4_^+^, suggesting that their capacity for assimilating ammonium is more effective than that of other, higher plants. In addition, the site-directed mutagenesis of PhGDH2 at positions Gly^193^ and Thr^361^ was important for its catalytic activity, and their expression levels were significantly increased under abiotic stresses. The findings allow for an improved understanding of the glutamic acid biosynthetic pathway, which is crucial for improving the quality of *P. haitanensis*.

Tannin-based extracts have been shown to possess natural phytocomplexes with health benefits, and may be used as feed supplements. Mattioli et al. have indicated that tannin-based extracts from *Castanea sativa* Mill. wood and *Schinopsis balansae* Engl. Quebracho Colorado hardwood contain hydrolysable tannins and condensed tannins, respectively, and these extracts can induce spontaneous intestinal contractility effects by regulating tone and food bolus progression [12]. The study proposes that the chemical compositions of these extracts could provide a foundation for future investigations of chemical compound classes for their use in the control of contractility within the pharmaceutical or agriculture sectors, as well as their possible potential in combining different combinations of *Castanea sativa* Mill. wood and *Schinopsis balansae* Engl. hardwood extracts for synergistic effects.

## 6. Conclusions

In summary, this Special Issue enhances our knowledge on the potential of biomolecules derived from diverse types of natural products that are present in the form of extracts, formulations, and chemical compounds. Together the research articles and reviews from this Special Issue can provide useful insights on the applications of biomolecules in pharmaceutical and agricultural industries.
